# The disproportionate excess mortality risk of COVID-19 in younger people with diabetes warrants vaccination prioritisation

**DOI:** 10.1007/s00125-021-05404-8

**Published:** 2021-02-16

**Authors:** Andrew P. McGovern, Nick J. Thomas, Sebastian J. Vollmer, Andrew T. Hattersley, Bilal A. Mateen, John M. Dennis

**Affiliations:** 1 Institute of Biomedical & Clinical Science, University of Exeter Medical School, Royal Devon & Exeter Hospital, Exeter, UK; 2 Diabetes and Endocrinology, Royal Devon and Exeter NHS Foundation Trust, Exeter, UK; 3 Alan Turing Institute, London, UK; 4 Department of Statistics, University of Warwick, Coventry, UK; 5 Institute of Health Informatics, University College London, London, UK

**Keywords:** COVID-19, Death, Diabetes, Vaccination

*To the Editor:* Diabetes has consistently been shown to independently increase the risk of poor coronavirus disease-2019 (COVID-19) outcomes [1–3]. Rather than a simple additive effect of diabetes and age-related risk, recent large studies suggest a more complex relationship, with a disproportionately higher excess relative mortality risk in younger people with diabetes compared with older people with diabetes [1, 3, 4]. Better understanding of the interaction between age and diabetes in the context of COVID-19 will further inform the complex prioritisation decisions around COVID-19 vaccination [5].

To explore this relationship, we triangulated the evidence on heterogeneity of diabetes effect by age on COVID-19 mortality from large population-based and critical care-based studies. Two UK population-based studies (OpenSAFELY [*n* = 17,278,392; 8.8% with diabetes] and QCOVID [*n* = 6,083,102; 7.0% with diabetes]) have previously reported adjusted age-specific hazard ratios for COVID-19-related mortality risk associated with diabetes [1, 4]. OpenSAFELY reported these stratified by recent HbA_1c_ measurements as recorded in primary care (</≥58 mmol/mol [</≥7.5%] or not available), but not by diabetes type. QCOVID reported age-specific hazard ratios for type 2 diabetes by sex, but did not report age-specific values for type 1 diabetes (see electronic supplementary material [ESM] Methods). The overall 90-day COVID-19-related mortality rate was 0.06% in OpenSAFELY (study period: 1 February 2020–6 May 2020) and the 97-day COVID-19-related mortality rate was 0.07% in QCOVID (derivation cohort study period: 24 January 2020–30 April 2020).

Building on our previous analysis in the critical care setting [3], we also examined adjusted age-specific hazard ratios associated with type 2 diabetes in people with severe COVID-19 using the COVID-19 Hospitalisation in England Surveillance System (CHESS) cohort (*n* = 19,256 individuals admitted to critical care in England; 18.3% with type 2 diabetes; see ESM Methods). In this cohort, the 30-day in-hospital mortality rate was 26.4% (see ESM Table 1 for age-stratified mortality rates).

To aid interpretability of our findings, based on the work of Spiegelhalter [6, 7], we translated hazard ratio estimates into ‘COVID-age’, which represents the additional years of COVID-19 mortality risk added to an individual’s chronological age if diabetes is present. Full details of our approach are provided in the ESM Methods. The study was reviewed and approved by the Warwick BSREC (BSREC 119/19-20).

The additional COVID-19 mortality risk associated with diabetes is, in terms of COVID-age, markedly higher in younger than older people (Fig. 1). This reflects the higher relative risk of COVID-19-related mortality associated with diabetes in younger age groups (hazard ratios for diabetes >5 in adults under 50 years of age in population-based studies). Population-based and critical care-based estimates are similar, despite differences in setting, time period and adjustments for confounders between studies. For a person aged 40 years with diabetes, additional mortality risk is equivalent to around 20 years of chronological age, meaning that mortality risk is similar to that of a 60-year-old person without diabetes. For a person aged 70 years with diabetes, the additional mortality risk from diabetes is equivalent to an additional 8 years of age, so their COVID-age is 78 years (based on QCOVID data).

**Fig. 1:**
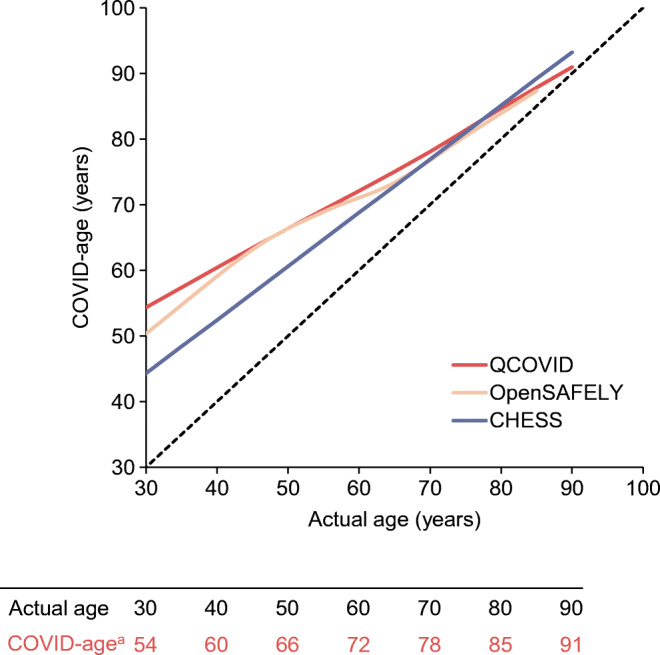
The additional years of COVID-19 mortality risk added to an individual’s chronological age if diabetes is present (‘COVID-age’) compared with actual age (dotted black line) in people aged 30–90 years. Data are from two large UK population-based studies (OpenSAFELY [*n*=17,278,392] and QCOVID [*n*=6,083,102]) and a national database of critical care patients in England (COVID-19 Hospitalisation in England Surveillance System [CHESS] cohort [*n*=19,256]). Underlying data are reported in ESM Table 2. ^a^COVID-age estimates are from QCOVID data

Clearly, considering only age and diabetes status when assessing COVID-19-associated risks (both mortality and in general) is an oversimplification. Multiple additional factors, including BMI, diabetes duration, glycaemic control, diabetes type and existing complications are known to further modify individual COVID-19 risk [8, 9]. Whilst patient-level risk incorporating these multiple factors can be calculated [10] (and is preferable for informing individuals of their COVID-19 risk), this is not practical for population-level vaccine rollout. The time-critical nature of population COVID-19 vaccination necessitates pragmatic group-level prioritisation, which is the approach initiated by governments thus far [11, 12].

Whilst the absolute risk of COVID-19-related mortality in younger people with diabetes is still not as high as that of the elderly, vaccine prioritisation approaches should not simply consider absolute mortality risk. Younger people are disproportionately impacted in terms of life years lost and are of working age, which puts them at potentially higher risk of exposure. These factors should be considered, alongside the excess relative COVID-19 mortality risk in younger people with diabetes, to ensure that they are appropriately prioritised for vaccination.

## Supplementary information

ESM 1 (PDF 372 kb)

## Data Availability

CHESS data cannot be shared publicly as it was collected by Public Health England as part of their statutory responsibilities, which allows them to process patient confidential data without explicit patient consent. Data utilised in this study were made available through an agreement between the University of Warwick and Public Health England. Individual requests for access to CHESS data are considered directly by Public Health England (contact via covid19surv@phe.gov.uk).

## Abbreviation

COVID-19: Coronavirus disease-2019
