# Supplementary material for: The disproportionate excess mortality risk of COVID-19 in younger people with diabetes warrants vaccination prioritisation
Source: Diabetologia. 2021 Feb 16;64(5):1184–6. doi: 10.1007/s00125-021-05404-8 (PMC7885981; doi:10.1007/s00125-021-05404-8)
Supplement: Supplementary file 1 — (PDF 372 kb) [file 125_2021_5404_MOESM1_ESM.pdf]

Supplementary Material for: The disproportionate excess mortality risk of COVID-19 in younger people with diabetes warrants vaccination prioritisation

Authors: Andrew P. McGovern, Nicholas J. Thomas, Sebastian J. Vollmer, Andrew T Hattersley, Bilal A. Mateen, John M. Dennis

ESM Methods

Study datasets

OpenSAFELY and QCOVID population based studies of COVID-19 related mortality

Both OpenSAFELY [n=17,278,392, 8.8% with diabetes] and QCOVID [n=6,083,102, 7.0% with diabetes] report age-specific hazard ratios for the adjusted effect of diabetes on COVID-19 related mortality in supplementary material.[1-3] OpenSAFELY provided these stratified by age category (18-39, 40-49, 50-59, 60-69, 70-70, 80+) and recent blood glucose control (recent HbA1c as recorded in primary care </≥58 mmol/mol [</≥7.5%], or not available [respectively 60.4%, 28.3%, and 11.3% of people with diabetes]), but did not stratify by diabetes type. For our analysis, we extracted the weighted mean hazard ratio at the mid-point of each category using the relative frequencies of each HbA1c defined subgroup. QCOVID provide estimates only for type 2 diabetes separately in males and females, by continuous age (modelled as a 3 3 fractional polynomial). For our analysis we extracted hazard ratios for type 2 diabetes at 10 year intervals from age 30-90, and applied Spiegalhalter’s formula with a Hazard ratio per year of higher age of 1.1.

CHESS cohort of patients admitted to critical care with COVID-19 in England.

Details of the CHESS cohort (n=19,256 admitted to critical care in England, 18.3% with type 2 diabetes) have been previously reported.[4, 5] In the critical care setting, we observed an approximately log-linear association for all-cause mortality by age for 30-day all-cause mortality after hospital admission, with an attenuated mortality increase of approximately 3.4% per year in comparison to the population-based studies. As we had access to the individual-level data for this cohort a type 2 diabetes by continuous age interaction term was fitted in an adjusted Cox regression model (adjusted for age, sex, ethnicity, obesity, and other major comorbidities (chronic respiratory disease, asthma, chronic heart disease, hypertension, immunosuppression, chronic neurological disease, chronic renal disease, and chronic liver disease), with hazard ratio outputs for type 2 diabetes from age 30-90 (10 year intervals) then translated into COVID-age estimates using Spiegalhalter’s approach but with a hazard ratio per year of age of 1.034.

Use of 'effective age' to communicate the additional years of mortality risk associated with a specific risk factor such as diabetes

Spiegelhalter,[6] draws on the work of Brenner et al,[7] to demonstrate that the presence of a risk factor such as diabetes can be expressed in terms of the number of years lost/gained if two conditions are filled. The first is the standard assumption of proportional hazards in Cox regression. The second conditions is that hazard increases exponentially with age (i.e. that each year increases the risk by a fixed percentage), which is the cases for all-cause mortality in the population.[6] Spiegelhalter has recently demonstrated that a very similar (although slightly steeper) exponential increase applies for COVID-19 mortality, with the average annual risk of death increasing by approximately 10% per year from age 35 (Hazard ratio per year of age 1.1).[8]

Given these two conditions appear to be satisfied for COVID-19 mortality, hazard ratios for a specific risk factor can be translated into effective age, the additional years of COVID-19 mortality risk associated with the presence of the risk factor, using the following procedure:[6]

(1) Assume the instantaneous risk of an event (h) $= e^{a+bx+ct}$, where e^{b} is the hazard ratio associated with a unit increase in the risk factor (x) and e^{c} is the hazard ratio associated with a year of ageing (chronological age = t).

(2) To calculate the additional years of mortality risk (t) which is equivalent to a unit change in the risk factor, the two risks are made equivalent: $e^{b} = e^{ct}$

(3) Therefore the additional age equivalent additional risk for a unit change in risk factor is given by; t = b/c

When considering the additional years of COVID-19 mortality risk associated with diabetes, b is log(hazard ratio for diabetes) obtained from the Cox regression models. The hazard ratio for COVID-19 mortality associated with diabetes varies with age. Therefore our estimates for the additional years of COVID-19 mortality risk associated with diabetes are stratified by age.

We adopt this approach and present our results in this more easily communicable manner we term 'COVID-age', representing the additional years of mortality risk associated with the presence of diabetes across people at all different ages (see Figure 1 of article). Estimates are triangulated across OpenSAFELY, QCOVID, and CHESS.

References

[1] Williamson EJ, Walker AJ, Bhaskaran K, et al. (2020) Factors associated with COVID-19-related death using OpenSAFELY. Nature 584(7821): 430-436. 10.1038/s41586-020-2521-4

[2] Post-publications supplement to Williamson, E.J., Walker, A.J., Bhaskaran, K. et al. OpenSAFELY: factors associated with COVID-19 death in 17 million patients. Nature (2020). https://doi.org/10.1038/s41586-020-2521-4. Available at: https://opensafely.org/assets/age_interactions_report_v1.2.pdf. Accessed 25.11.2020.

[3] Clift AK, Coupland CAC, Keogh RH, et al. (2020) Living risk prediction algorithm (QCOVID) for risk of hospital admission and mortality from coronavirus 19 in adults: national derivation and validation cohort study. BMJ 371: m3731. 10.1136/bmj.m3731

[4] Dennis JM, Mateen BA, Sonabend R, et al. (2020) Type 2 Diabetes and COVID-19–Related Mortality in the Critical Care Setting: A National Cohort Study in England, March–July 2020. Diabetes Care: dc201444. 10.2337/dc20-1444

[5] Dennis JM, McGovern AP, Vollmer SJ, Mateen BA (2020) Improving Survival of Critical Care Patients With Coronavirus Disease 2019 in England: A National Cohort Study, March to June 2020. Critical Care Medicine Online First

[6] Spiegelhalter D (2016) How old are you, really? Communicating chronic risk through 'effective age' of your body and organs. BMC Medical Informatics and Decision Making 16(1): 104. 10.1186/s12911-016-0342-z

[7] Brenner H, Gefeller O, Greenland S (1993) Risk and rate advancement periods as measures of exposure impact on the occurrence of chronic diseases. Epidemiology (Cambridge, Mass) 4(3): 229-236. 10.1097/00001648-199305000-00006

[8] Spiegelhalter D (2020) Use of "normal" risk to improve understanding of dangers of covid-19. BMJ 370: m3259. 10.1136/bmj.m3259

ESM Table 1: Absolute 30 day in-hospital mortality, by type 2 diabetes status, in patients admitted to critical care in England (n=19,256, CHESS dataset).

Age group	No type 2 diabetes			Type 2 diabetes		
	No. admitted	No. died	% died	No. admitted	No. died	% died
18-44	1786	95	5.3%	190	24	12.6%
45-54	2039	230	11.3%	429	69	16.1%
55-64	2814	547	19.4%	848	239	28.2%
65-74	2978	852	28.6%	872	338	38.8%
75-84	3334	1170	35.1%	718	300	41.8%
85-99	2781	1006	36.2%	467	207	44.3%

ESM Table 2: Estimated increase in effective age associated with the presence of diabetes. Effective age + chronological age = COVID-age as presented in Figure 1.

a) QCOVID

**Chronological Age (years)**	**30**	**40**	**50**	**60**	**70**	**80**	**90**
**Effective Age due to Presence of Diabetes (years)**							
**Female**	24.0	19.0	14.6	10.3	6.7	3.9	1.0
**Male**	24.7	21.6	17.9	13.6	9.2	5.4	1.0
**Overall**	24.3	20.4	16.4	12.1	8.1	4.7	1.0

b) OpenSAFELY

**Chronological Age (years)**	**18-39**	**40-49**	**50-59**	**60-69**	**70-79**	**80+**
**Effective Age due to Presence of Diabetes (years)**						
**HbA1c <58 mmol/mol (7.5%)**	19.7	17.3	12.4	6.7	3.6	0.9
**HbA1c ≥58 mmol/mol (7.5%)**	23.6	20.4	16.4	11.8	7.4	3.9
**HbA1c not recorded**	11.0	15.7	14.1	5.9	8.3	4.9
**Overall**	20.4	18.1	13.9	8.3	5.4	2.3

c) CHESS

**Chronological Age (years)**	**30**	**40**	**50**	**60**	**70**	**80**	**90**
**Effective Age due to Presence of Diabetes (years)**							
**Overall**	14.6	12.7	10.9	9.1	7.3	5.4	3.6
